# Medical News and Miscellany

**Published:** 1888-10

**Authors:** 


### Medical News and Miscellany.

DR. J. W. McNEILL, of Valley Mills, is reported very sick.

DR. WM. BARRY, of Abilene, is confined to his bed with an abscess of the hip.

. CHANGE OF FIRM. The firm of Drs. Irion & Cochrane, Montgomery, Texas, is changed to Irion & Young.

DR. A. B. DENTON, of Weldon, died recently, date not known. He was a member of the Texas State Medical Association.

DR. Q. C. SMITH, of Austin, delivered an address last week before the Travis County Teachers' Institute, on "Hygiene of the School Room."

Dr. T. J. TYNER, of this city, last week made a cataract extraction|without iridectomy, removing capsule and all, intact; a very rare feat in ophthalmic surgery.

DR. LEE T. MADDOX, of Birdston, Navarro county, died on the 10 inst., at an advanced age. He was a member of the Physicians' Mutual Benefit Association. Assessment will be issued in due time.

THE *Statesman* tells of the killing of a doctor at Brownsboro, under peculiar circumstances. He was lying, drunk, in the street, and a citizen passing by, shot him, claiming that "he thought it was a dog!"

"Mr. 'Pottecary," said an urchin, running in in breathless haste and a ragged straw-hat, "Mommer say send her *fi* cents woth sreet spets niter, *fi* cents woth sreet spets camphire, and—and *fi* cents woth sreet spets of turkentime!"

ERRATA.—In Dr. Wooten's article for "cocti," read "cocci." In Dr. Cunningham's, read 1887 for 1877. In list of members P. M. B. A., for "Hors" read "Dr. Hons," (page 169); on page 166, in East Line Medical Society, for "editorial papers" read "elaborate papers."

---

REMOVALS.—Dr. M. R. Denman, Elmo to Luling; Dr. J. A. White, Bartlett to Prairie Plains; Dr. J. W. Hargas, Cotulla to Carrizo Springs; Dr. F. M. D. Hill, Webberville to Cat Spring; Dr. H. A. Hughes, Glenrose to Phœnix, Arizona; Dr. D. B. McGee, Jardin to Hinckley; Dr. D. V. Spring Lyons to Milano.

---

DR. W. F. BLUNT has been removed by State Health Officer Dr. Rutherford, from charge of the Galveston Quarantine Station, and Dr. Fred. K. Fisher, formerly Q. O. at Aransas Pass, has been appointed in his stead. No charges were made against Dr. Blunt, so far as we are informed, except "incompatability of views" with the chief.

---

THE "IMMACULATE ONE HUNDRED," or "THE" Association of the ONLY Simon-pure-and-genuine-unadulterated "American physicians" had a love-feast at Washington lately. "Billings on Museums," was the Presidential address. They had a fine time, they say—and all felt, mutually delighted with each other, and with the success of their meeting.

---

DR. LONG'S WIDOW.—Mrs. Caroline Long, of San Antonio, widow of the celebrated Dr. Crawford Long, of Athens, Ga., discoverer of chloroform, was killed near Comfort, Texas, in a wreck on the Aransas Pass road, Saturday, 22nd of September. The deceased lady was the mother-in-law of the well-known Texas editor, Col. John L. Bartow.

---

ANNUAL BANQUET.—The famous West Texas Medical Society will hold their annual reunion October 31st, and wind up with a grand banquet as usual. We acknowledge the courtesy of an invitation to be present, and regret our inability to do so. The efficient secretary, Dr. D. Berry, takes the occasion of this invitation to enclose us $2, and the name of a new subscriber, for which we make our bow.

---

RAILROAD CORPORATIONS, AND THEIR DUTIES AND RESPONSIBILITIES TO SOCIETY,'' is the name of an admirable paper by ex-State Health Officer R. M. Swearingen, which was read at the Austin District Medical Society on September 20th, and voted to be published in this, the Society's official journal. It will appear in the next issue. Send in your orders now for extra copies; it will have a big run.

---

A PAPER by Dr. C. M. Ramsdell of Lampasas, read before the Section on Electro-Therapeutics at the Galveston meeting T. S. M. A., will appear in our next. Also a paper by Dr. F. A. Schmidt, of Schulenburg, which was intended for this issue, but unavoidably omitted. We have a number of good papers on hand—read recently at different society meetings, and they will be published with the discussions that followed them.

---

DIED—In Rusk, Cherokee county, Tex., Oct. 3, 1888, Dr. T. F. F. Jameson, aged 61,—a native of Wilcox Co., Ala., and born Sept. 27, 1827. Dr. Jameson graduated in medicine in Lexington, Kentucky, in '53, and in Augusta, Ga., in '54. He practiced in Alabama till 1859, when he removed to Cherokee county, Texas, where he resided up to the date of his death. His son, Dr. W. G. Jameson, of Rusk, survives him. Dr. Jameson was one of the oldest and most respected physicians in East Texas.

---

THE NEW ORLEANS MEDICAL AND SURGICAL JOURNAL gives our State Health officer, Dr. Rutherford, ''Hail Columbia'' for quarantining Texas against New Orleans, on mere heresay, as charged by the *Journal;* and says, as Texas was represented in the conference of State Boards, at which an agreement was made to place implicit confidence in each other, and when one State wanted facts as regards epidemic diseases, appeal should be made to the Board of the suspected State, Dr. Rutherford had violated the pledge by ignoring the Health Board of Louisiana. The *N. O. M. and S. Journal*, having one big fight on hand, (with the State Medical Society), should be careful how it flings stones at our plucky little S. H. O.;—he hits back sometimes.

SENATOR PLUMB has introduced a bill in Congress, offering a reward of $100,000 for a "sure cure" for yellow fever! (When a "sure cure" for death itself is found, he will get it.) Mr. Radam, a florist of Austin, arguing that what will kill insects on plants will kill "microbes," and assuming that microbes are like insects, and that they cause yellow fever, has concocted a mixture which he calls "Microbe Killer." He offered it to the senator, with a note, gravely informing him that he "would enter the contest for the $100,000 prize. As a specimen of monumental, gigantic, adamantic cheek, the offer is entitled to first premium. Mr. R. says he "wrote to Dr. Hamilton" also, offering to cure, right out of hand, (we suppose, any and all cases of) yellow, (blue, black, green, or any other) fever, (or anything else, death not even excepted,)—and he actually complained, *in the newspapers*, that Dr. Hamilton *didn't answer his letters!* Hamilton ought to be ashamed of himself.

GEO. PINNEY, of Evergreen, Door county, Wis., is out with by far the most extensive catalogue of Evergreens and Timber Trees and Tree Seeds ever published in this country. It contains price lists and descriptions of over one hundred varieties. He offers to send a copy to any person asking for it.

---

NUMBER 10, of The Physicians' Leisure Library, is at hand. These little books are issued monthly by Geo. S. Davis, of Detroit, Mich. The present number treats of "Diseases of the Male Uretha," by Fessenden N. Otis, M. D.

AT THIS SEASON, when cotton is being marketed, the doctors make their collections for the year's work,—if ever. We avail ourselves of the fact to notify all our subscribers whose subscription has expired, of the expiration, and to solicit a renewal. We have sent bills to all, and will be *delighted* to have all renew. We do not want any of them to consider it "a dun," and get mad,—but,—if nobody paid in advance, we'd have a hard time running an 80-page journal C. O. D. from the press;—and the new subscribers are also availing themselves of the above fact, to invest in "*the best and most useful* JOURNAL published in the medical language," as one waggish friend puts it. Send 'em along, gentlemen; the more subscribers, the better JOURNAL.

---

"YOUR JOURNAL improves all the time. I do not see how any doctor in Texas can get along without it," is what one subscriber writes; and another says, "Enclosed find $5. I can't do without the JOURNAL. I am anxious to do all I can to support it,—will write for its pages soon. Hoping you are well, and the JOURNAL prosperous, I am, yours," etc.

And still another, "Of all the journals that come to my desk each month, it is the best, most useful and entertaining; it is superb."

*Per contra:* One gentleman got as mad as a *wet hen* because we notified him that his subscription had expired, and most courteously solicited a renewal. He sent four bits, and said, "Stop my JOURNAL." *She's stopped!*

---

THE INTERNATIONAL JOURNAL OF SURGERY AND ANTISEPTICS for October, contains an excellent likeness of the late Dr. C. R. Agnew, of New York. Subscription to the journal is $1 a year; single copies 30 cts. Address Dr. F. King, manager, P. O. Box 581, New York.

---

WE ARE REQUESTED TO ANNOUNCE that Messrs. Mariani & Co. have removed their offices and warerooms from 127 Fifth Avenue, to 52 W. Fifteenth Street, New York, where they "will be happy to receive the members of the medical profession," and whence "all corresponcence will have their prompt attention."

